# Phorbol Esters from Jatropha Meal Triggered Apoptosis, Activated PKC-δ, Caspase-3 Proteins and Down-Regulated the Proto-Oncogenes in MCF-7 and HeLa Cancer Cell Lines

**DOI:** 10.3390/molecules170910816

**Published:** 2012-09-10

**Authors:** Ehsan Oskoueian, Norhani Abdullah, Syahida Ahmad

**Affiliations:** 1 Department of Microbiology, Faculty of Biotechnology and Biomolecular Sciences, Universiti Putra Malaysia, Serdang 43400, Selangor, Malaysia; Email: ehs424@yahoo.com; 2 Agriculture Biotechnology Research Institute of Iran (ABRII)-East and North-East Branch, Mashhad 91735, Iran; 3 Department of Biochemistry, Faculty of Biotechnology and Biomolecular Sciences, Universiti Putra Malaysia, Serdang 43400, Selangor, Malaysia; Email: syahida@biotech.upm.edu.my; 4 Institute of Tropical Agriculture, Universiti Putra Malaysia, Serdang 43400, Selangor, Malaysia

**Keywords:** phorbol esters, Jatropha meal, apoptosis, anti proliferation, gene expression, Western blot, DNA fragmentation

## Abstract

Jatropha meal produced from the kernel of *Jatropha curcas* Linn. grown in Malaysia contains phorbol esters (PEs). The potential benefits of PEs present in the meal as anticancer agent are still not well understood. Hence, this study was conducted to evaluate the cytotoxic effects and mode of actions of PEs isolated from Jatropha meal against breast (MCF-7) and cervical (HeLa) cancer cell lines. Isolated PEs inhibited cells proliferation in a dose-dependent manner of both MCF-7 and HeLa cell lines with the IC_50_ of 128.6 ± 2.51 and 133.0 ± 1.96 µg PMA equivalents/mL respectively, while the values for the phorbol 12-myristate 13-acetate (PMA) as positive control were 114.7 ± 1.73 and 119.6 ± 3.73 µg/mL, respectively. Microscopic examination showed significant morphological changes that resemble apoptosis in both cell lines when treated with PEs and PMA at IC_50_ concentration after 24 h. Flow cytometry analysis and DNA fragmentation results confirmed the apoptosis induction of PEs and PMA in both cell lines. The PEs isolated from Jatropha meal activated the PKC-δ and down-regulated the proto-oncogenes (c-Myc, c-Fos and c-Jun). These changes probably led to the activation of Caspase-3 protein and apoptosis cell death occurred in MCF-7 and HeLa cell lines upon 24 h treatment with PEs and PMA. Phorbol esters of Jatropha meal were found to be promising as an alternative to replace the chemotherapeutic drugs for cancer therapy.

## 1. Introduction

*Jatropha curcas* Linn. (family *Euphorbiaceae*) is receiving a lot of attention nowadays due to the demand for seed oils for the biodiesel industry [1]. In addition, the ethnopharmacological investigation indicates the usefulness of this plant in traditional medicine to cure various infectious diseases [2]. The notable antioxidant, anticancer and anti-inflammatory activities of the extracts obtained from the root, latex and seed [3,4] and the antimicrobial activity of root and stem have been reported [5]. The seeds contain 50%–60% oil, which is a potential feedstock for the biodiesel industry [6]. The byproduct which is called Jatropha meal contains bioactive peptides [7] and phytochemicals including phenolics, phytic acid, trypsin inhibitors, lectins, saponins and phorbol esters (PEs) [8].

**Figure 1:**
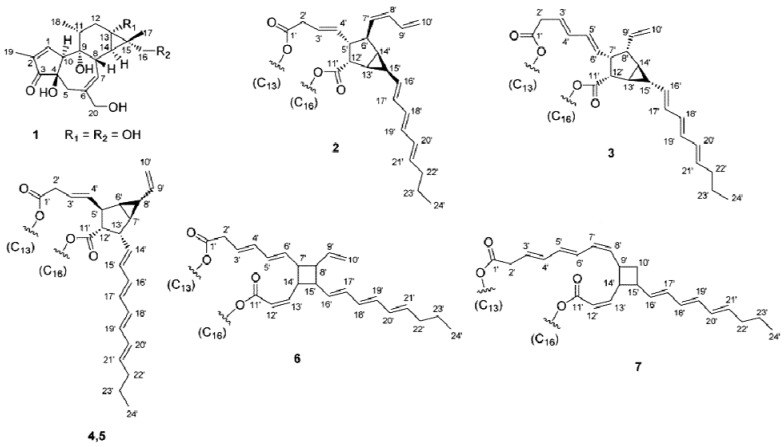
The characteristics of phorbol esters from *J. curcas* seeds [10].

The presence of phorbol esters was first reported in croton oil [9]. They are polycyclic compounds, where two hydroxyl groups on neighboring carbon atoms are esterified with fatty acids. The PEs present in *J. curcas* seeds have been characterized by Hass *et al.* [10] as shown in Figure 1. All six isolated PEs possessed the same diterpene moiety identified as 12-deoxy-16-hydroxyphorbol. The dicarboxylic acid moieties of **2**–**5** contained a bicyclo[3.1.0]hexane unit, and those of **6** and **7** appeared to have a cyclobutane unit. Moreover, the compounds **4** and **5** were reported to be C-8 epimers.

Phorbol esters are known as activators of protein kinase C (PKC). It is believed that, the number and position of functional groups in the structure of PEs probably determines the type of activated PKC isozymes leading to the activation of different pathways. Thus, upon exposure to the PEs, various phenomena such as tumorgenesis, apoptosis, inflammation and survival of the cells have been reported. For instance, Goel *et al.* [9] reported the potential of some naturally occurring PEs from *Croton tiglium*, *Jatropha gossypifolia* and* Ostodes paniculata* to inhibit tumor development, human immunodeficiency virus (HIV) replication and leukemia. The wide variety of PEs’ potentials provide new opportunities for research on treatments of cancer, inflammation, cardiovascular diseases, Alzheimer’s symptoms and acquired immunodeficiency syndrome [11].

To date, the medicinal properties of PEs from Jatropha meal have not been fully elucidated. Concomitant to the PEs present in other plant materials, PEs from Jatropha meal may have cytotoxic properties and could be an alternative source of chemotherapeutic drugs. The disadvantage of chemotherapeutic drugs is the occurrence of side effects and the development of drug resistance after a certain period. Thus, application of alternative therapies using natural resources should be explored. Therefore this research was conducted to evaluate the cytotoxic effects and mode of actions of PEs isolated from Jatropha meal against breast (MCF-7) and cervical (HeLa) cancer cell lines.

## 2. Results and Discussion

### 2.1. Isolation of Phorbol Esters

**Figure 2:**
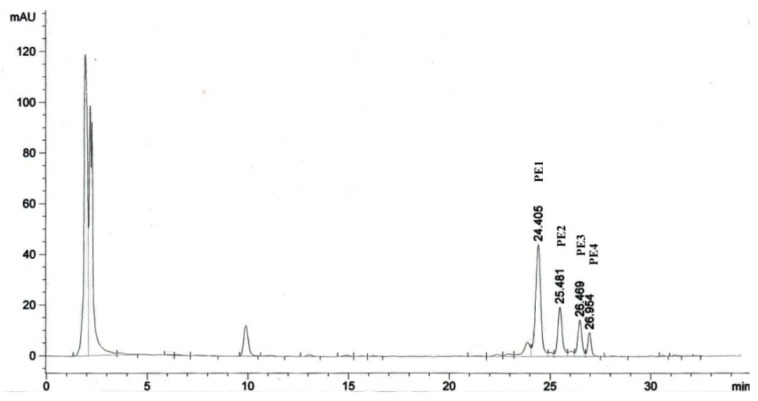
HPLC chromatogram of the PEs present in Jatropha meal.

The high performance liquid chromatography (HPLC) analysis (Figure 2) illustrated that the Jatropha meal PEs appeared in four peaks which were labelled as PE1, PE2, PE3 and PE4. Their retention times were similar to those of the PEs reported by Makkar *et al.* [12] and Li *et al.* [13] as described in Section 3.2. Hass *et al.* [10] have also characterised the PEs of *J. curcas* seed and confirmed the presence of the six PEs as shown in Figure 1. Although the number of the PEs were six but in the present analysis they appeared in four peaks (Figure 2), this could be possibly due to the similar molecular weight of some of the PEs present in Jatropha meal.

The concentrations of the isolated PEs used in this study were expressed as equivalents of the standard phorbol-12-myristate 13-acetate (PMA). The total yield of PEs isolated from Jatropha meal was 3 mg PMA equivalent/g dry matter of Jatropha meal. Based on the results observed, the proportions of PE1, PE2, PE3 and PE4 were 57.5, 20.6, 13.9 and 7.8% of the total PEs, respectively. At this stage, due to the lack of information on the biological activities of PEs from Jatropha meal, this study was focused on the cytotoxic properties of the PEs. The PEs were pooled, thus the biological activities observed in this experiment corresponded to the total PEs present in Jatropha meal.

### 2.2. Proliferation Assay

**Figure 3:**
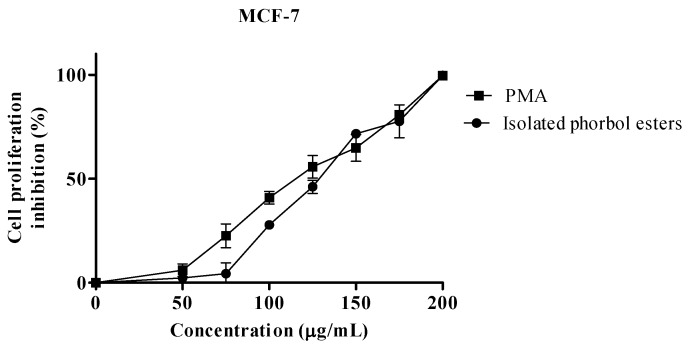
Percentage of cell proliferation inhibition of isolated PEs and PMA on MCF-7 cell line. Values represent mean ± SEM of three replicates.

**Figure 4:**
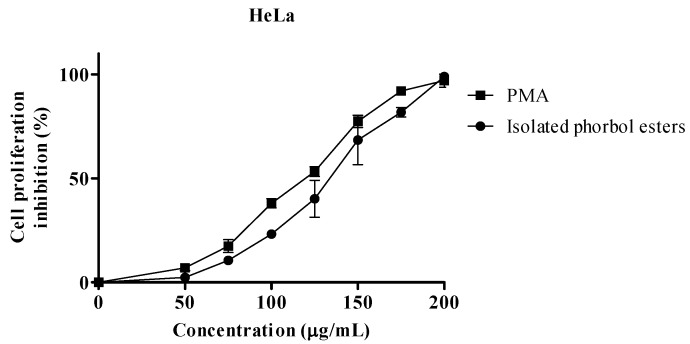
Percentage cell proliferation inhibition of isolated PEs and PMA on HeLa cell line. Values represent mean ± SEM of three replicates.

The anti proliferative activities of isolated PEs and PMA as positive control in MCF-7 and HeLa, are shown in Figure 3 and Figure 4 respectively. Isolated PEs and PMA inhibited the cells proliferation in a dose-dependent manner.

The IC_50_ values presented in Table 1 show similar concentrations (*p* > 0.05) of PEs and PMA to inhibit the proliferation of 50% of the cells for MCF-7 and HeLa cells. The difference in IC_50_ values between PMA and PEs for each cell line indicated the possible dissimilarity in the structures of PEs isolated from Jatropha meal.

**Table 1:** IC_50_ concentration of isolated PEs and PMA in MCF-7 and HeLa cell lines.

	IC_50_ µg/mL		
	MCF-7	HeLa	S.E.M
**PEs**	128.6	133.0	1.69
**PMA ^1^**	114.7	119.6	2.16

^1^ PMA: Phorbol-12-myristate 13-acetate; Analyses were done in triplicates.

### 2.3. Microscopic Examination

**Figure 5:**
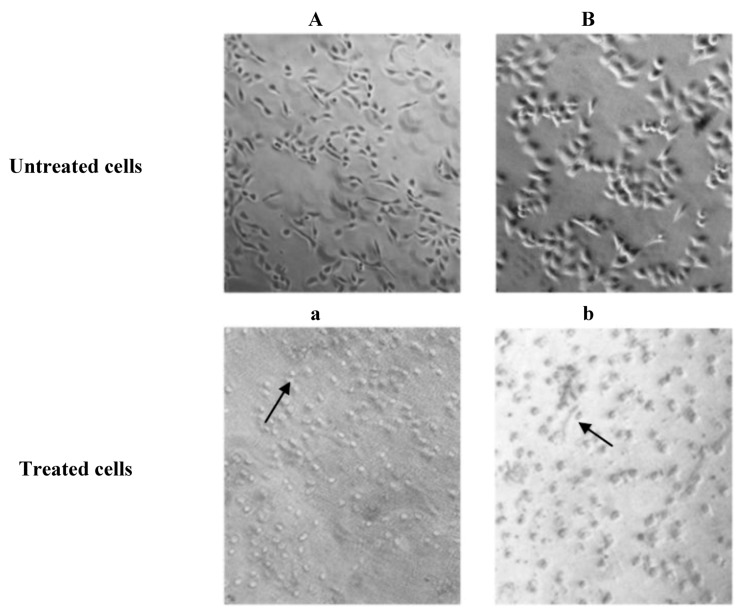
Morphological changes observed in different cell lines upon treatment with isolated PEs at IC_50_ concentration (**a**–**b**) after 24 h incubation examined by light microscopy at 200× magnification. MCF-7(**A**,**a**), HeLa (**B**,**b**). The arrows show the apoptotic bodies and destructed cells.

The results of morphological changes visualized in different cell lines upon treatment with isolated PEs at IC_50_ concentration (a–b) after 24 h incubation are presented in Figure 5. Significant morphological changes, as well as detachment and destruction of cells were observed in both types of cancer cells after 24 h treatment with PEs.

According to these microscopic observations (Figure 5), the cell damage resembles apoptosis as cell walls were not intact and apoptotic bodies were seen. Both cancer cell lines displayed death upon treatment with the PEs and PMA at IC_50_ concentration after 24 h incubation. The present study indicated that PEs isolated from Jatropha meal initially disrupt the cell-substream adhesion, without immediate loss of viability, subsequently cells detachment and finally death with apoptosis characteristics in MCF-7 and HeLa cell lines. These results support the finding of Avila *et al.* [14] and Bond *et al.* [15] who demonstrated a dose-dependent toxic action of PMA on pancreatic cancer cell lines. These authors also suggested that the growth inhibitory of PMA is associated with an increase in apoptosis which contributes to its anti tumor effects.

### 2.4. Analysis of Apoptosis by Flow Cytometry

The results of flow cytometry analysis are presented in Table 2. These results showed that cell viability of MCF-7 and HeLa cell lines with initial values of 98.1 and 98.7% decreased significantly (*p* < 0.05) to 29.7 and 31.5% upon treatment with PEs and to 26.4 and 29.5% upon treatment with PMA, respectively. The MCF-7 and HeLa cell lines showed 30.3 and 25.9% apoptotic cells upon treatment with PEs, while cells treated with PMA showed significantly (*p* < 0.05) higher values at 35.4% for MCF-7 cell line. Although, the PEs appeared to be less active as compared to the PMA in induction of apoptosis, the percentage of dead cells indicated no significant difference between the cells treated with PEs and PMA. The difference in the potential of PMA and PEs in induction of apoptosis could probably due to the numbers or the position of functional groups present in the PEs structures. In addition, the comparison of apoptotic cell values in both cell lines indicated that the MCF-7 cells showed the apoptosis symptoms earlier than HeLa cells in the presence of PEs. The flow cytometry result also confirmed that PEs isolated from Jatropha meal and also PMA induced apoptosis cell death upon 24 h exposure.

**Table 2:** Percentage of viable, apoptotic and dead cells analysed by flow cytometry.

	MCF-7 Cells (%)			HeLa Cells (%)			S.E.M
	Untreated	PEs-treated	PMA-treated	Untreated	PEs-treated	PMA-treated	
**Viable**	98.1 ^a^	29.7 ^b^	26.4 ^c^	98.7 ^a^	31.5 ^b^	29.5 ^b^	2.78
**Apoptotic**	1.5 ^d^	30.3 ^b^	35.4 ^a^	2.3 ^d^	25.9 ^c^	28.8 ^bc^	2.28
**Dead**	2.2 ^d^	52.4 ^ab^	55.1 ^a^	2.6 ^d^	46.8 ^c^	48.9 ^bc^	3.47

At least 13,000 cells were analysed by flow cytometry; Means with different superscripts within rows are significantly different (*p* < 0.05); Analyses were done in triplicates.

### 2.5. DNA Fragmentation Assay

**Figure 6:**
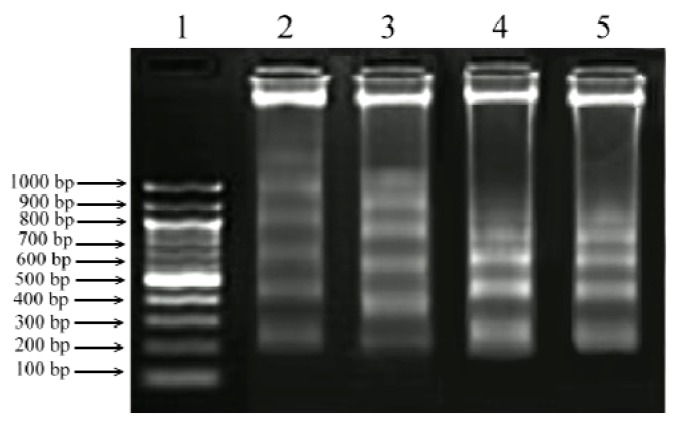
DNA fragmentation induced by isolated PEs and PMA in MCF-7 and HeLa cancer cell lines at IC_50_ concentration. The extracted DNA was run on 2% agarose gel and the image was documented using Bio-Rad Gel documentation system. Lane 1: 1 kb DNA ladder; Lane 2: MCF-7+PEs; Lane 3: MCF-7+PMA; Lane 4: HeLa+PEs; Lane 5: HeLa+PMA.

DNA fragmentation is a natural phenomenon that takes place in cells undergoing apoptosis. As shown in Figure 6 the isolated PEs and PMA induced nucleosome-sized DNA fragmentation. The presence of DNA cleavage bands in cells treated with PEs indicated the similar cytotoxic effect of PEs to that of PMA. This result was in agreement with Day *et al.* [16] who observed changes in morphological features, apoptosis and endonuclease digestion of genomic DNA after 24 h incubation in human prostate adenocarcinoma cells (LNCaP) treated with PMA.

### 2.6. Gene Expression Analysis

The expression analyses of proto-oncogenes including c-Myc, c-Fos, and c-Jun in MCF-7 and HeLa cells upon treatment with isolated PEs and PMA are shown in Table 3. The expression of c-Myc gene in MCF-7 and HeLa cell lines showed significant down-regulation with the value of −2.6 and −2.3 fold changes upon treatment with PEs and −3.2 and −3.6 fold changes upon treatment with PMA, respectively. Similarly, the expression of c-Jun gene in MCF-7 and HeLa cell lines was significantly down-regulated with the value of −1.3 and −1.7 fold changes after treatment with PEs and −1.7 and −2.2 fold changes upon treatment with PMA, respectively. The c-Fos gene in both cell lines was also significantly down-regulated with the value of −2.1 and −2.5 fold changes while treated with PEs and −3.2 and −3.5 fold changes after treatment with PMA, respectively.

**Table 3:** Fold-changes in the expression levels of c-Myc, c-Jun and c-Fos genes in MCF-7 and HeLa cell lines upon treatment with PEs and PMA.

Down-regulated genes	MCF-7 Cells				HeLa Cells			
	PEs	*p* ^1^	PMA	*p*	PEs	*p*	PMA	*p*
**c-Myc**	−2.6	0.02	−3.2	0.03	−2.3	0.03	−3.6	0.04
**c-Jun**	−1.3	0.04	−1.7	0.02	−1.7	0.04	−2.2	0.03
**c-Fos**	−2.1	0.03	−3.2	0.04	−2.5	0.02	−3.5	0.05

^1^ *p value*: The genes with *p* < 0.05 are considered significantly down-regulated as compared to the un-treated cells.

These genes are known as proto-oncogenes and their expression levels in the cancer cells are abnormally higher than normal cells. The proto-oncogenes are often involved in signal transduction pathway. In fact, the c-Myc gene is responsible for cell growth and proliferation, differentiation and apoptosis, while c-Fos/c-Jun complexes interact with AP-1 site on the promoter to regulate the expression of various genes involved in everything from proliferation and differentiation to defence against invasion and cell damage. The down-regulation of proto-oncogenes in this study may be mediated through the PKC family since Hatton *et al.* [17] has shown the activation of PKC is the earliest response of the cells to the presence of PMA and this activation affected the expression of downstream genes including proto-oncogenes. In line with the result of this study, Udou *et al.* [18] has also reported the role of PMA in activation of PKC which resulted in down-regulation of c-Jun gene in glandular epithelial cells.

### 2.7. Western Blot Assay

**Figure 7:**
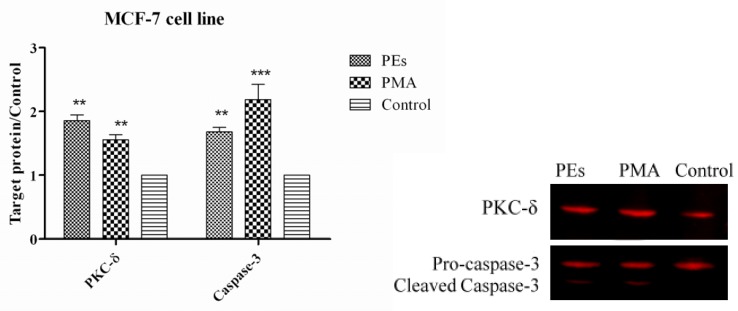
Expression of PKC-δ and Caspase-3 proteins in treated and un-treated MCF-7 cell line. Cells were treated with isolated PEs from Jatropha meal and PMA at the IC_50_ concentration incubated for 24 h. Equal amounts of total cellular protein of treated and un-treated cells were subjected to Western blot analyses for PKC-δ, Caspase-3 and GAPDH protein expression. All values represent mean ± standard error from three independent experiments, *** *p* ≤ 0.001 and ** p ≤ 0.01 indicate significant difference compared to the untreated control.

**Figure 8:**
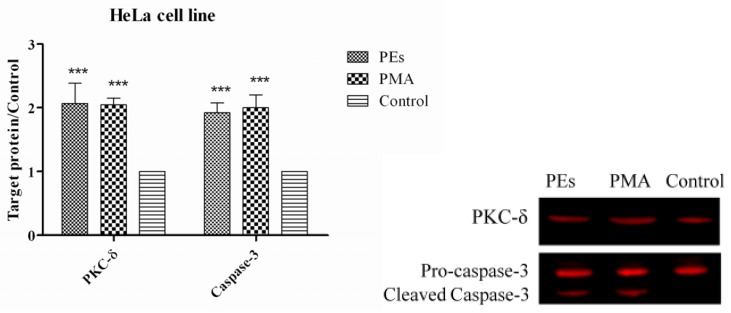
Expression of PKC-δ and Caspase-3 proteins in treated and un-treated HeLa cell line. Cells were treated with isolated PEs from Jatropha meal and PMA at the IC_50_ concentration incubated for 24 h. Equal amounts of total cellular protein of treated and un-treated cells were subjected to Western blot analyses for PKC-δ, Caspase-3 and GAPDH protein expression. All values represent mean ± standard error from three independent experiments, *** *p* ≤ 0.001 and ** p ≤ 0.01indicate significant difference compared to the untreated control.

As shown in Figure 7 and Figure 8, PKC-δ protein was significantly (*p* < 0.01) over-expressed in both cell lines treated with isolated PEs and PMA. The results also indicated significant (*p* < 0.01) over-expression and cleavage of Caspase-3 protein in both cell lines as one of the feature of apoptosis. In fact, PEs are known as activator of PKC and their binding to PKC is the first step in activation of PKC. This binding is saturable and occurs through specific interactions within the C1 domain in the regulatory region of the PKC molecule [11], However, the response of the cell could vary depending on the types of activated PKC. In most systems, PKC-α, ε and ι act as anti-apoptotic kinases, whereas PKC-θ, μ and δ act as pro-apoptotic kinases [19]. In line with this result, several researchers reported the pro-apoptotic effect of PMA in different cell lines [20,21]. The over-expressed PKC-δ in this study confirmed the pro-apoptotic effects of PEs upon 24 h incubation, concomitant to the results of flow cytometry.

Caspases comprise a family of different cysteine proteases that are synthesized as inactive zymogens and are activated by proteolysis [22]. The activation of Caspase-3 upon different apoptotic stimuli is dependant on various initiator pathways. Basically, the generation of pro-apoptotic signals in death receptors and even mitochondria could also activate an initiator of upstream caspase, which usually possesses a long NH_2_-terminal prodomain such as found in caspases-8, -9 and -10. These initiator caspases can activate the Caspase-3 and results in apoptotic execution [23]. Laouar *et al.* [24] have also reported that the activation of PKC in the presence of PMA led to activation of caspase cascade proteins and finally apoptosis in human myeloid HL-60 leukemia cells. Consequently, the apoptosis observed in this study could be the result of PKC-δ activation by PEs and PMA which resulted in down-regulation of proto-oncogenes including c-Myc, c-Fos and c-Jun genes. Down-regulation of these genes could be the reasons of activation of Caspase-3 and apoptosis execution.

Phorbol 12-myristate 13-acetate as an activator of PKC isozymes may promote tumor formation [9] or apoptosis [25]. Day *et al.* [16] suggested that activation of a PMA-inducible kinase(s) mediates apoptosis of androgen-sensitive prostate cells by means of an intracellular pathway that may involve the transient activation of the early response transcription factors NGFI-A and c-Fos, whereas, Fujii *et al.* [26] reported that PMA induced apoptosis in prostate cancer cells through over-expression of PKC-δ. In contrast, Park [27] concluded that the PMA is not only a tumor promoter, but can also induce apoptosis in gastric cancer cells through activation of PKC and the activation of serine protease(s) and Caspase-3/CPP32. Indeed, the multiplicity effects of PEs on biological systems are associated with the type of PEs, type of cell, time of exposure and other experimental conditions which can affect either pro-apoptotic or anti-apoptotic activities.

## 3. Experimental

### 3.1. Plant Materials

The *Jatropha curcas* L. plant was collected from the farm of Faculty of Agriculture, Universiti Putra Malaysia (GPS location of 3°0'26.91"N latitude and 101°42'13.24"E longitude) for identification by Mr. Shamsul Khamis and its voucher specimen (SK1764/2010) was deposited in the Phytomedicinal Herbarium, Institute of Bioscience, Universiti Putra Malaysia, Serdang, Selangor, Malaysia. Upon confirmation of the plant, the mature *J. curcas* seeds were collected from the farm, air dried and dehulled. The kernel were ground by using a mechanical grinder followed by oil extraction with a Soxhlet apparatus, using petroleum ether (boiling point of 40–60 °C), for 16 h [28]. Defatted kernel (Jatropha meal) was air dried at room temperature and kept in screw cap bottle at −20 °C.

### 3.2. Phorbol Esters Isolation

Phorbol esters from Jatropha meal were isolated according to Makkar and Becker [12] and Li [13]. Briefly, the sample (4 g) was extracted with methanol at least for five times with pestle and mortar and then the methanol evaporated by rotary evaporator. The crude methanolic extract was dissolved in 5 mL of methanol and an aliquot was loaded on a Waters Alliance 2695 Separations Module (Waters, Milford, MA, USA) high-performance liquid chromatography system (HPLC) equipped with a Waters 996 Photodiode Array Detector, and a reverse-phase, C_18_ LiChrospher 100, 250 × 4 mm I.D and 5 μm pore size column (Agilent Technologies, Waldbronn, Germany). The separation was performed using a gradient elution with solvents comprising deionized water and acetonitrile [12]. The absorbance was read at 280 nm and peaks were observed at 24.4, 25.5, 26.5 and 26.9 min. Phorbol esters were carefully collected using a fraction collector (Waters) at the retention times stated above. The collected fractions were pooled and freeze dried. Isolated PEs were redissolved in dimethyl sulfoxide and injected to HPLC to check the purity and concentration. The concentration of the isolated PEs used in this study was expressed as equivalent to the standard, phorbol-12-myristate 13-acetate (PMA).

### 3.3. Cell Lines and Cell Culture

Two cancer cells including human breast cancer cells (MCF-7 ATCC: HTB-22) and human cervical adenocarcinoma (HeLa ATCC: CCL-2) were purchased from the American Type Culture Collection (ATCC). Cells were grown as monolayers in a T-75 cm^2^ culture flask. The Dulbecco’s Modified Eagle Medium (DMEM) was supplemented with 2.0 g/L sodium bicarbonate and 10% fetal bovine serum. The cell cultures were maintained in a humidified atmosphere of 5% CO_2_ at 37 °C and were harvested when they reached 80% confluency.

### 3.4. Proliferation Assay

Cell proliferation was determined using 3-(4,5-Dimethylthiazol-2-yl)-2,5-Diphenyltetrazolium Bromide (MTT) according to Sharif *et al.* [29]. Monolayers of the cells (5 × 10^3^/100 μL) were grown in 96-well microtitre plates and treated with the isolated PEs from 200 μg to 50 μg/mL by serial dilution. After 24 h incubation at 37 °C, cells proliferation assay was determined based on the reduction of MTT by the mitochondrial dehydrogenase of intact cells into an insoluble purple formazan product. Phorbol-12-myristate 13-acetate was used as a positive control in the present study.

### 3.5. Microscopic Examination

Cells were cultured and treated at IC_50_ concentration with isolated PEs as well as positive control. Morphological apoptotic changes were examined after 24 h incubation and photographed using a phase-contrast microscope.

### 3.6. Analysis of Apoptosis by Flow-Cytometry

A fluorescent-activated cell sorting (FACS) analysis was performed to detect apoptosis. The MCF-7 and HeLa cells were seeded at the density of l × 10^6^ cells per 75 cm^2^ flask and cultured for 24 h in DMEM. Once confluent, the media were removed and fresh media containing PEs and PMA at the CC_50_ concentration were added. The treated cells were then incubated at 37 °C in 5% CO_2_ for 24 h. The FITC Annexin V Apoptosis Detection Kit I (BD Biosciences Pharmingen, San Diego, CA, USA) was used to stain the cell, following the manufacturer’s instructions. The stained cells were monitored by flow cytometry (FACS-Canto II BD Biosciences) and the data were analyzed using Diva software (BD Biosciences).

### 3.7. DNA Fragmentation Assay

Different cell lines were treated at IC_50_ concentration of PEs and incubated for 24 h. Culture cells were harvested and washed by PBS and pelleted by centrifugation at 300 ×*g* for 10 min. DNA was extracted from cells using extraction buffer containing Na_2_HPO_4_ and citric acid followed by addition of RNase and proteinase K according to Darzynkiewicz and Juan [30]. Extracted DNA was applied to 2% agarose gel and electrophoresed at 50 V for 3 h and the gels were stain with ethidium bromide.

### 3.8. Gene Expression Analyses

Cells were cultured and treated as mentioned in section 3.6. The media was removed after 24 h and cells were washed twice with cold phosphate buffered saline (PBS). Cells were scrapped and the RNA of cells were extracted by using RNasey Mini kits (Qiagen, Valencia, CA, USA) according to the protocol recommended by the manufacturer. The concentration, integrity and size distribution of total RNA extracted was checked. The reverse transcriptase PCR (RT-PCR) was performed using Maxime RT Premix kit (iNtRON Biotechnology, Sungnam, Korea) according to the protocol of the manufacturer. Real-time PCR assays were conducted on a BioRad CFX 96 real-time PCR thermocycler (Bio-Rad, Hercules, CA, USA) using iQ SYBR Green Supermix (Bio-Rad). The primers for c-Jun, c-Fos, c-Myc and GAPDH are presented in Table 4.The optimized PCR reaction condition for c-Fos, c-Jun, c-Myc and GAPDH genes were as follows: 94 °C for 5 min (1×), then 94 °C for 20 s, then 60 °C for 20 s and 72 °C for 25 s (40×). The expression of studied genes were normalized to GAPDH expression according to Vandesompele *et al.* [31]. Data from the real-time PCR reactions were analyzed using CFX manager software version 2 (Bio-Rad Laboratories). All real-time PCR amplifications were performed in triplicate.

**Table 4:** Characteristics of the PCR primers sets used for gene expression analysis.

Targeted gene	Forward	Reverse	Reference
c-Myc	tgcgtgaccagatccc	cgcacaagagttccgta	[32]
c-Jun	cttcaacccaggcgcgctgagca	gtctgaggctcctccttcagggcct	[33]
c-Fos	tgatgacctgggcttcccag	caaagggctcggtcttcagc	[33]
GAPDH	ccggatcgaccactacctgggcaac	gttccccacgtactggcccaggacca	[34]

All directions are from 5' to 3'.

### 3.9. Western Blotting

Expression of protein kinase C-δ (PKC-δ) and Caspase-3 proteins were assessed by Western blot analysis. Briefly, cells were trypsinized, harvested and washed three times with cold PBS. Then the cells were lysed in 100 μL of Lysis buffer (0.5% Triton X-100, 2 mM EDTA in 20 mM Tris-HCl pH 7.5) containing 10 μL/mL of Protease Inhibitor Cocktail (ProteoBlock Protease Inhibitor Cocktail, Fermentas, Glen Burnie, MD, USA) at 4 °C. Cells were then sonicated and incubated in ice for 20 min and supernatant collected after centrifugation at 14,000 × *g* for 30 min. Protein concentration was determined in supernatant using the Protein Assay Kit (Bio-Rad) and 20 μg of protein was denatured by incubation at 95 °C for 5 min and subjected to electrophoresis using Tris-glycine polyacrylamide gel. Proteins were transferred electrophoretically to a PVDF membrane using the Hoefer Semi-Dry Transfer Unit, (Hoefer Scientific Instruments, San Francisco, CA, USA). After electroblot transfer of the protein, membranes were washed twice and incubated with Odyssey Blocking Buffer (LI-COR, Lincoln, NE, USA) for 1 h at room temperature with rocking to block non-specific antibody binding. Then the membrane was incubated overnight with a 1:1000 dilution of PKC-δ (PAB18258 Abnova), 1:1000 dilution of GAPDH (Thermo Scientific MA1-4711) and 1:500 dilution of Caspase-3 (Biorbyt orb10237) primary antibodies. Membrane was washed with 0.05% PBST (phosphate buffer saline and Tween 20) three times for 5 min. For detection with the Odyssey imaging system, a 1:10000 dilution of the IRDye 800 CW Goat Anti-Rabbit Secondary Antibody or -IRDye 680 Goat Anti-Mouse Secondary Antibody was used. Membrane was washed with 0.05% PBST three times for 5 min. The membrane was dried and visualized using the Odyssey Infrared Imaging System (LI-COR, Lincoln, NE, USA) and Odyssey software was used to determine the intensity of the proteins band.

### 3.10. Statistical Analysis

Statistical analysis was conducted using GLM procedure [35] using a complete randomized design following the model: Yi = µ + Ti + ei, where µ is the mean value, Ti is the treatment effect and ei is the experimental error, respectively. Differences in LSD were considered significant at *p* < 0.05. GraphPad Prism 5 software (GraphPad Software Inc., San Diego, CA, USA) was used for all the statistical analyses in Western blotting.

## 4. Conclusions

The PEs isolated from Jatropha meal activated the PKC-δ and down-regulated the proto-oncogenes (c-Myc, c-Fos and c-Jun). These changes led to the activation of Caspase-3 protein and apoptosis cell death occurred in MCF-7 and HeLa cell lines upon 24 h treatment with PEs. The results showed that isolated PEs behaved similarly to PMA in induction of apoptosis. Phorbol esters of Jatropha meal were found to be a promising alternative to replace other chemotherapeutic drugs for cancer therapy based on the two cell lines studied. Further investigation on the response of other cancer cells to PEs isolated from Jatropha meal is recommended.

## References

[1] Thomas R., Sah N., Sharma P. (2008). Therapeutic biology of *Jatropha curcas*: A mini review. Curr. Pharm. Biotechnol..

[2] Aiyelaagbe O.O., Hamid A.A., Fattorusso E., Taglialatela Scafati O. (2011). Cytotoxic activity of crude extracts as well as of pure components from Jatropha species, plants used extensively in african traditional medicine. Evid. Based Complementary Altern. Med..

[3] Oskoueian E., Abdullah N., Ahmad S., Saad W.Z., Omar A.R., Ho Y.W. (2011). Bioactive compounds and biological activities of *Jatropha curcas* L. kernel meal extract. Int. J. Mol. Sci..

[4] Oskoueian E., Abdullah N., Saad W.Z., Omar A.R., Ahmad S., Kuan W.B., Zolkifli N.A., Hendra R., Ho Y.W. (2011). Antioxidant, anti-inflammatory and anticancer activities of methanolic extracts from *Jatropha curcas* L. J. Med. Plants Res..

[5] Namuli A., Abdullah N., Sieo C.C., Zuhainis S.W., Oskoueian E. (2011). Phytochemical compounds and antibacterial activity of *Jatropha curcas* Linn. extracts. J. Med. Plants Res..

[6] Murugesan A., Umarani C., Subramanian R., Nedunchezhian N. (2009). Bio-diesel as an alternative fuel for diesel engines—A review. Renew. Sust. Energ. Rev..

[7] Devappa R.K., Makkar H.P.S., Becker K. (2010). Nutritional, biochemical, and pharmaceutical potential of proteins and peptides from Jatropha: Review. J. Agric. Food Chem..

[8] Oskoueian E., Abdullah N., Saad W.Z., Omar A.R., Puteh M.B., Ho Y.W. (2011). Anti-nutritional metabolites and effect of treated *Jatropha curcas* kernel meal on rumen fermentation *in vitro*. J. Anim. Vet. Adv..

[9] Goel G., Makkar H.P.S., Francis G., Becker K. (2007). Phorbol esters: Structure, biological activity, and toxicity in animals. Int. J. Toxicol..

[10] Haas W., Mittelbach M. (2002). Novel 12-deoxy-16-hydroxyphorbol diesters isolated from the seed oil of *Jatropha curcas*. J. Nat. Prod..

[11] Kazanietz M.G. (2005). Targeting protein kinase C and “non-kinase” phorbol ester receptors: Emerging concepts and therapeutic implications. BBA-Proteins Proteom..

[12] Makkar H.P.S., Siddhuraju P., Becker K. (2007). Methods in Molecular Biology: Plant Secondary Metabolites.

[13] Li C.Y., Devappa R.K., Liu J.X., Lv J.M., Makkar H.P.S., Becker K. (2009). Toxicity of *Jatropha curcas* phorbol esters in mice. Food Chem. Toxicol..

[14] Avila G.E., Zheng X., Cui X.X., Ryan A.D., Hansson A., Suh J., Rabson A.B., Chang R.L., Shih W.J., Lin Y. (2005). Inhibitory effects of 12-*O*-tetradecanoylphorbol-13-acetate alone or in combination with all-trans retinoic acid on the growth of cultured human pancreas cancer cells and pancreas tumor xenografts in immunodeficient mice. J. Pharmacol. Exp. Ther..

[15] Bond J.A., Gescher A.J., Verschoyle R.D., Lemoine N.R., Errington R., Wiltshire M., Smith P.J., Wynford Thomas D. (2007). Cytotoxic action of phorbol esters on human pancreatic cancer cells. Int. J. Cancer.

[16] Day M., Zhao X., Wu S., Swanson P., Humphrey P. (1994). Phorbol ester-induced apoptosis is accompanied by NGFI-A and C-fos activation in androgen-sensitive prostate cancer cells. Cell Growth Differ..

[17] Hatton J.P., Gaubert F., Cazenave J.-P., Schmitt D. (2002). Microgravity modifies protein kinase C isoform translocation in the human monocytic cell line U937 and human peripheral blood T-cells. J. Cell. Biochem..

[18] Udou T., Hachisuga T., Tsujioka H., Kawarabayashi T. (2004). The role of c-jun protein in proliferation and apoptosis of the endometrium throughout the menstrual cycle. Gynecol. Obstet. Inves..

[19] Oh J.I., Chun K.H., Joo S.H., Oh Y.T., Lee S.K. (2005). Caspase-3-dependent protein kinase C delta activity is required for the progression of Ginsenoside-Rh2-induced apoptosis in SK-HEP-1 cells. Cancer Lett..

[20] Harborne J.B., Williams C.A. (2000). Advances in flavonoid research since 1992. Phytochemistry.

[21] Chen J., Giridhar K.V., Zhang L., Xu S., Wang Q.J. (2011). A protein kinase C/protein kinase D pathway protects LNCaP prostate cancer cells from phorbol ester-induced apoptosis by promoting ERK1/2 and NF-κB activities. Carcinogenesis.

[22] Cheftel J.C., Hefnawy M. (2011). Emerging risks related to food technology. Advances in Food Protection.

[23] Lai J.M., Hsieh C.L., Chang Z.F. (2003). Caspase activation during phorbol ester-induced apoptosis requires ROCK-dependent myosin-mediated contraction. J. Cell Sci..

[24] Laouar A., Glesne D., Huberman E. (2001). Protein kinase C-β, fibronectin, α5β1-integrin, and tumor necrosis factor-α are required for phorbol diester-induced apoptosis in human myeloid leukemia cells. Mol. Carcinogen..

[25] Hofmann J. (2004). Protein kinase C isozymes as potential targets for anticancer therapy. Curr. Cancer Drug Tar..

[26] Fujii T., Garcia-Bermejo M.L., Bernabo J.L., Caamano J., Ohba M., Kuroki T., Li L., Yuspa S.H., Kazanietz M.G. (2000). Involvement of protein kinase C (PKC) in phorbol ester-induced apoptosis in LNCaP prostate cancer cells. J. Biol. Chem..

[27] Park I.C., Park M.J., Rhee C.H., Lee J.I., Choe T.B., Jang J.J., Lee S.H., Hong S.I. (2001). Protein kinase C activation by PMA rapidly induces apoptosis through caspase-3/CPP32 and serine protease(s) in a gastric cancer cell line. Int. J. Oncol..

[28] Association of Official Analytical Chemists (AOAC) (2000). Official Methods of Analysis.

[29] Sharif R., Ghazali A., Rajab N., Haron H., Osman F. (2008). Toxicological evaluation of some Malaysian locally processed raw food products. Food Chem. Toxicol..

[30] Darzynkiewicz Z., Juan G., Henderson D.S. (1999). Selective extraction of fragmented DNA from apoptotic cells for analysis by gel electrophoresis and identification of apoptotic cells by flow cytometry. DNA Repair Protocols: Eukaryotic Systems.

[31] Vandesompele J., De Preter K., Pattyn F., Poppe B., Van Roy N., De Paepe A., Speleman F. (2002). Accurate normalization of real-time quantitative RT-PCR data by geometric averaging of multiple internal control genes. Genome Biol..

[32] Oxelmark E., Roth J.M., Brooks P.C., Braunstein S.E., Schneider R.J., Garabedian M.J. (2006). The cochaperone p23 differentially regulates estrogen receptor target genes and promotes tumor cell adhesion and invasion. Mol. Cell. Biol..

[33] Hu D.G., Mackenzie P.I. (2009). Estrogen receptor α, fos-related antigen-2, and c-Jun coordinately regulate human UDP glucuronosyltransferase 2B15 and 2B17 expression in response to 17β-estradiol in MCF-7 cells. Mol. Pharmacol..

[34] Rangatia J., Vangala R.K., Treiber N., Zhang P., Radomska H., Tenen D.G., Hiddemann W., Behre G. (2002). Downregulation of c-Jun expression by transcription factor C/EBPα is critical for granulocytic lineage commitment. Mol. Cell. Biol..

[35] SAS (2003). SAS, Statistical Analysis Institute (Version 9.1.3).

